# Synergistic Effect of Surface Plasmonic particles and Surface Passivation layer on ZnO Nanorods Array for Improved Photoelectrochemical Water Splitting

**DOI:** 10.1038/srep29907

**Published:** 2016-07-21

**Authors:** Yichong Liu, Xiaoqin Yan, Zhuo Kang, Yong Li, Yanwei Shen, Yihui Sun, Li Wang, Yue Zhang

**Affiliations:** 1 State Key Laboratory for Advanced Metals and Materials, School of Materials Science and Engineering, University of Science and Technology Beijing, Beijing 100083, China; 2 Civil and Environment Engineering school, University of Science and Technology Beijing, Beijing 100083, China; 3 The Beijing Municipal Key Laboratory of New Energy Materials and Technologies, University of Science and Technology Beijing, Beijing 100083, China

## Abstract

One-dimensional zinc oxide nanorods array exhibit excellent electron mobility and thus hold great potential as photoanode for photoelelctrochemical water splitting. However, the poor absorption of visible light and the prominent surface recombination hider the performance improvement. In this work, Au nanoparticles and aluminium oxide were deposited onto the surface of ZnO nanorods to improve the PEC performance. The localized surface plasmon resonance of Au NPs could expand the absorption spectrum to visible region. Simultaneously, the surface of passivation with Au NPs and Al_2_O_3_ largely suppressed the photogenerated electron-hole recombination. As a result, the optimal solar-to-hydrogen efficiency of ZnO/Au/Al_2_O_3_ with 5 cycles was 6.7 times that of pristine ZnO, ascribed to the synergistic effect of SPR and surface passivation. This research reveals that the synergistic effect could be used as an important method to design efficient photoanodes for photoelectrochemical devices.

Hydrogen, as a clean and renewable energy resource, can be used to tackle the current energy crisis and environmental issues. Among extensive efforts to hydrogen generation, photoelectrochemical(PEC) water splitting attracts considerable attention as it integrats in the same device both solar energy collection and water electrolysis. Recently, metal oxides such as titanium dioxide(TiO_2_)^1^,^2^, zinc oxide(ZnO)^3^,^4^, hematite(Fe_2_O_3_)^5^,^6^ and cuprous oxide(Cu_2_O)^7^,^8^ have shown great potential as photoelectrodes in photoelectrochemical cells for hydrogen evolution. Among these semiconductor candidates, ZnO shows a unique crystalline structure, a direct wide band gap (3.37 eV), a large exciton binding energy (60 meV), excellent electron mobility (115-155 cm^2^·V^−1^·s^−1^) and environmental compatibility^9^,^10^,^11^,^12^. Recently the strategy of synergistic effect, such as three dimension/plasmonic nanoparticles and three dimension/narrow band gap semiconductor, has been proved to be an effective way to obtain efficient solar water splitting. For example, Bai *et al*. reported a novel ZnO NAs/RGO/ZnIn_2_S_4_ heterostructure as a photoanode for photoelectrochemical water splitting. The ZnIn_2_S_4_ acted as visible light sensitizers and RGO was used to facilitate electron transfer synergistically contributed to over 200% enhancement of photo-to-hydrogen efficiency compared to bare ZnO NAs^4^. However, there is few reports about the synergistic effect of SPR and surface passivation on ZnO nanorods array to improve photoelectrochemical water splitting.

Lately, to increase the light-harvesting efficiency, plasmonic Au-modified ZnO electrodes have been designed to enhance PEC performance^13^,^14^,^15^. For example, Wu *et al*. reported a novel matchlike zinc oxide (ZnO)/gold (Au) with tunable Au contents. The photocurrent density and the solar-to-hydrogen conversion efficiency of the matchlike ZnO/Au could reach 9.11 mA/cm^2^ and 0.48%, respectively, which were much higher than that of the pristine ZnO^16^. Wang *et al*. synthesized Au nanoparticles sensitized ZnO nanopencil arrays. The Au-ZnO nanopencil arrays yield a photocurrent of ~1.5 mA·cm^−2^ at 1 V versus Ag/AgCl, which showed a significant enhancement compared with Au-ZnO nanorods array^17^. Although the recombination of photoexcited electron-hole could be suppressed to some extent after deposition of Au nanoparticles, the surface charge recombination at the Au-uncovered ZnO surfaces is still remained.

The passivation layers by atomic layer deposition (ALD) have shown great potential in the photoelectric devices^18^,^19^,^20^. Al_2_O_3_ as a high-κ dielectric has exhibited excellent surface passivation for electrochemical energy conversion devices^21^,^22^,^23^, leading to reduce surface charge recombination. In the case of PEC water splitting, Al_2_O_3_ overlayer via ALD have been employed in Al_2_O_3_/TiO_2_^21^, Al_2_O_3_/Fe_2_O_3_^22^, and Al_2_O_3_/WO_3_^24^ photoanodes with enhanced PEC performance.

In this work, we verified the elevated PEC performance by introducing Au NPs and Al_2_O_3_ overlayer to ZnO nanorods array. The optimal thickness of Al_2_O_3_ overlayer was acquired through accurately controlling the number of ALD cycles. The results demonstrated the ZnO/Au/Al_2_O_3_ with 5 cycles (ZAA-5) delivered the best photocurrent density response compared to the ZnO/Au or pristine ZnO photoanode, respectively. Such enhancement was ascribed to the synergistic effect of SPR induced by Au NPs and surface passivation resulted from the Au NPs and Al_2_O_3_ overlayer.

## Results and Discussions

**Figure 1:**
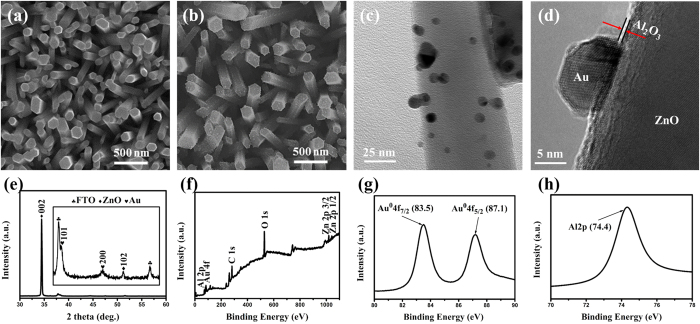
(**a**) Top view SEM image of pristine ZnO. (**b**) Top view SEM image of ZnO/Au. (**c**) A low-magnification TEM image and (**d**) a HRTEM image of ZnO/Au/Al_2_O_3_ (5cycles). (**e**) XRD patterns of the ZnO/Au/Al_2_O_3_ (5 cycles) on the FTO substrate. XPS spectra of ZnO/Au/Al_2_O_3_ (5 cycles): (**f**) wide scan, (**g**) Au 4f and (h) Al 2p.

Figure 1(a) showed a typical SEM image of the ZnO NRs array with a high density, well-aligned orientation and smooth surfaces. The average diameter and length of the nanorods were ~100 nm and ~3.5 μm, respectively (cross-sectional-view image shown in Figure S1(a) in the Supporting Information). Figure 1(b) showed that Au nanoparticles were uniformly distributed on the surface of each ZnO nanorod and the nanorods still maintained their vertically-oriented characteristics. Figure1(c) showed a HRTEM image taken near the edge of the ZnO nanorods, demonstrating a direct attachment of the Au nanoparticles onto the surface of ZnO nanorods. And the overlayer (labeled by a red arrow in Fig. 1(d)) indicated the amorphous characteristics of Al_2_O_3_ shell with an average thickness of about 0.56 nm. The compositions of ZAA-5 were further examined by energy-dispersive X-ray spectroscopy (EDS) measurements and their atomic ratios were calculated from the EDS spectra, as shown in Figure S1(b) and (c) in the Supporting Information.

X-ray diffraction spectrum in Fig. 1(e) was performed to reveal that all diffraction peak of ZnO nanorods array correspond to the standard diffraction of a wurtzite structure (JCPDS file No. 36-1451) with no impurity, and the intense peak at 2θ = 34.52° for samples showed the significant vertical orientation of ZnO nanorods array. The peaks centered at 2θ of 38.1° and 44.4°, which can be identified as characteristic peaks of (111) and (200) of Au nanocrystal (JCPDS file No. 04-784). However, no significant diffraction peaks of Al_2_O_3_ were observed probably due to the formation of the amorphous Al_2_O_3_ overlayer after the ALD process.

The XPS spectrum in Fig. 1(f) revealed the existence of Zn, O, Al and Au elements of ZnO/Au/Al_2_O_3_ sample. High-resolution spectra of Au and Al species were shown in parts (g) and (h) of Fig. 1, respectively. The two peaks centered at 83.5 eV and 87.2 eV in Fig. 1(g) can be attributed to Au 4f_7/2_ and Au 4f_5/2_, respectively. A lower binding energy of Au 4f_7/2_ was mainly due to electrons transfer from oxygen vacancies of ZnO to Au. The Al 2p spectrum in Fig. 1(h) was assigned a peak at 74.4 eV, corresponding to the typical position of pure Al_2_O_3_.

**Figure 2:**
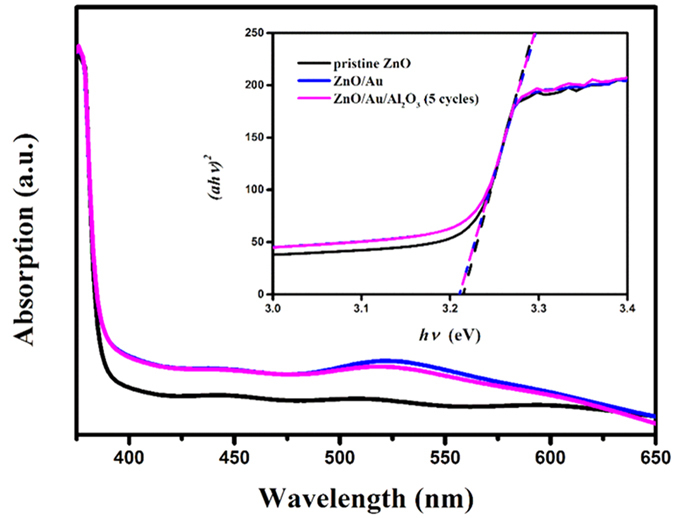
UV-Vis absorption spectra of pristine ZnO, ZnO/Au and ZnO/Au/Al_2_O_3_ (5 cycles). The inset is the corresponding (ahv)n vs hv curves of the three samples. Here, n evaluated to be two.

The UV-visible absorption spectra of as-synthesized photoanodes were measured and compared over a wavelength range from 370 nm to 650 nm shown in Fig. 2. The three photoanodes all exhibited a huge absorption in ultraviolet region because of the large band gap of ZnO. The optical band gap of ZnO was estimated to be about 3.21 eV based on the converted (*ahν*)^n^ versus *hν* (shown in the inset of Fig. 2), which was slightly lower than the previously reported band gap value of other groups^9^. This little deviation should be attributed to inevitable and ineffective carbon doping (shown in Figure S2 in the Supporting Information) during the synthesis process of the ZnO NRs array^25^. And there was no influence on the optical absorption edge in the UV region with the deposition of Au NPs and Al_2_O_3_ overlayer. As Au NPs were attached on the ZnO NRs, there was an absorption peak around 525 nm corresponding to the localized SPR effect of Au NPs, which was another convincing proof of the modification of Au NPs. The dielectric medium of Al_2_O_3_ overlayer resulted in the slightly red-shifted and small change of the intensity of the SPR peak.

**Figure 3:**
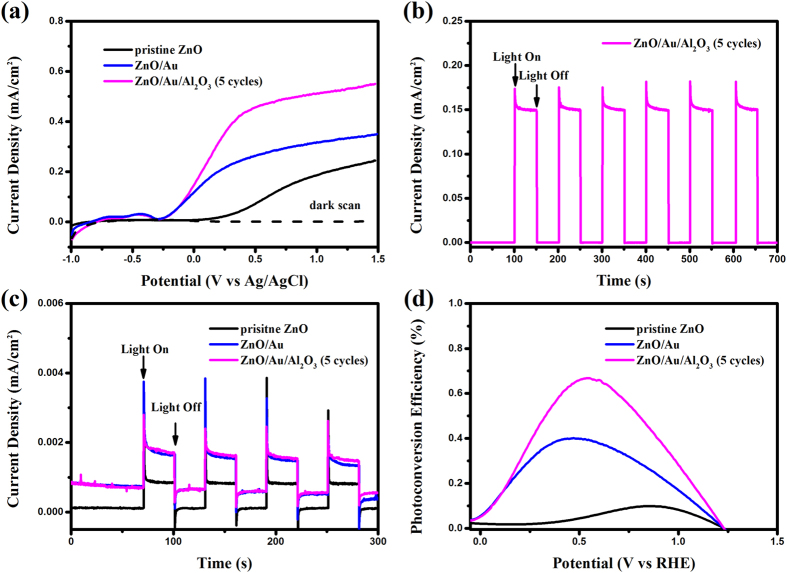
(**a**) LSV curves recorded for pristine ZnO, ZnO/Au and ZnO/Au/Al_2_O_3_ (5 cycles) photoanodes with a scan rate of 10 mV/s in the applied potentials from −1.0 to 1.5 V (vs Ag/AgCl) under AM 1.5 G simulated solar light. (**b**) Transient photocurrent density for ZnO/Au/Al_2_O_3_ (5 cycles) photoanodes measured at 0 V vs Ag/AgCl in 0.5 M Na_2_SO_4_ electrolyte (pH = 7). (**c**) Chronoamperometric I-t curves collected at 0.5 V vs Ag/AgCl for the three different photoanodes under visible light (>420 nm). (**d**) Photoconversion efficiency of the PEC cell with three different photoanodes as a function of the applied photential (vs RHE).

The PEC performance of as-synthesized various photoanodes were characterized through photoinduced I-V curves. As can be seen in Fig. 3(a), the pristine ZnO photoanode exhibited a very small background current density from −1 to 1.5 V (vs. Ag/AgCl) in the scale of 10^−3^ mA·cm^−2^. Under illumination, the pristine ZnO photoanode revealed a pronounced photocurrent density starting at 0.4 V and continued to increase to 0.2 mA·cm^−2^ at 1.5 V (vs. Ag/AgCl). After the modification of Au nanoparticles, the photocurrent density reached 0.3 mA·cm^−2^ at 1.5 V (vs. Ag/AgCl). This result demonstrated that the Au nanoparticles on the ZnO nanorods were beneficial to enhance the PEC performance, resulting from plasmonic-improved light absorption and surface passivation. With the deposition of Al_2_O_3_ overlayer, the ZAA-5 photoanode showed optimized photocurrent density in the whole potential window, and the saturated photocurrent density was as high as 0.55 mA·cm^−2^ attributed to the surface passivation resulting in the reduction of the recombination. Figure 3(b) showed chronoamperometry at 0 V vs. Ag/AgCl under chopped illumination conditions (1 sun, AM 1.5 G) for ZAA-5 photoanode. At 0 V vs. Ag/AgCl, a high and stable photocurrent density of was obtained, which showed consistent on/off photocurrent behaviors. Figure 3(c) showed the I-t curves of the three photoanodes under the illumination of visible light with λ > 420 nm. For the pristine ZnO, there was barely light response under visible light. In contrast, Au NPs modified ZnO NRs photoanode exhibited a significant photoresponse ascribed to the reason that introduction of Au NPs enhanced the visible light-harvesting efficiency via SPR effect. After the deposition of Al_2_O_3_ overlayer, there was no difference compared to ZnO/Au photoanode, which was in line with UV-Vis absorption spectra in Fig. 2.

Furthermore, the solar-to-hydrogen (STH) efficiencies (η) for PEC water splitting of such photoanodes were estimated using the following equation:


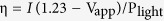


V_app_ is the applied external potential versus reversible hydrogen electrode (RHE). I is the externally measured current density at V_app_. P_light_ is the power density of the incident light. The potentials were measured versus the Ag/AgCl reference electrode and converted to the reversible hydrogen electrode (RHE) scale using the Nernst function:


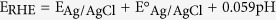


E_RHE_ is the converted potential versus RHE. E_Ag/AgCl_ is the external potential measured against the Ag/AgCl reference electrode. E^°^_Ag/AgCl_ is the standard electrode potential of Ag/AgCl reference electrode (0.1976 V versus RHE at 25 °C).

As shown in Fig. 3(d), the maximum STH efficiency of the pristine ZnO photoanode was 0.10% at an applied voltage of 0.88 V vs. RHE, whereas the maximum efficiency of the ZnO/Au photoanode reached 0.40% at an applied voltage of 0.47 V vs. RHE under the same conditions. Obviously, the ZAA-5 exhibited the optimal STH efficiency (0.67% at 0.55 V vs. RHE), 1.7 times and 6.7 times that of ZnO/Au and pristine ZnO photoanodes, respectively. The enhanced PEC activities benefited from its unique feature of the ZAA-5 composite structure.

**Figure 4:**
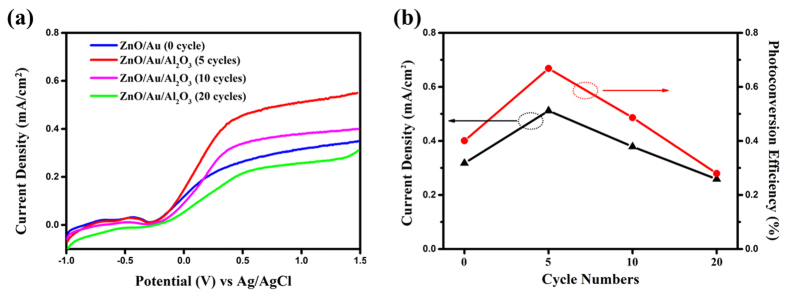
(**a**) LSV curves recorded for the ZnO/Au/Al_2_O_3_ photoanodes with various cycles. (**b**)Plot of the obtained photocurrent density and photoconversion efficiency as function of the ZnO/Au/Al_2_O_3_ photoanodes with various cycles at a scan rate of 10 mV/s under 40 mW/cm^2^ light illumination.

To confirm the effect of the thickness of Al_2_O_3_ on the photocurrent enhancement, PEC performance of photoanodes with various cycles were carried out. Figure 4(a) showed the LSV curves of the as-prepared ZnO/Au/Al_2_O_3_ (X cycles, X = 0, 5, 10 and 20) photoanodes. The changes of the photocurrent densities measured at 1.0 V (vs. Ag/AgCl) against the deposition cycles were shown in Fig. 4(b). The photocurrent densities were enhanced as the cycles increased from 0 to 5cycles and then decreased with further increase of the cycles. The photocurrent density and STH efficiency reached the maximum (0.512 mA/cm^2^ and 0.67%), as a result of the reduction of the recombination. However, excessive deposition of the insulation overlayer would possibly hinder the transmission of electron resulting in photocurrent decay.

**Figure 5:**
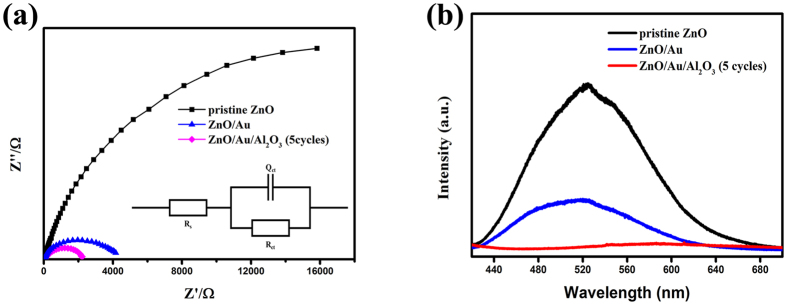
(**a**) Nyquist plots of electrochemical impedance spectra of the different photoanodes measured at 0 V (vs Ag/AgCl) under illumination. The inset is the equivalent circuit employed to fit the Nyquist plots. (**b**) Photoluminescence (PL) spectra of the diffierent photanodes.

Electrochemical impedance spectroscopy was employed under illumination at 0 V vs. Ag/AgCl to investigate the charge transfer kinetics at the photoanode/electrolyte surface (shown in Fig. 5(a)). Obviously, the arc diameter of the ZZA-5 photoanode was the smallest, while the arc diameter of the ZnO/Au photoanode was much smaller than that of the pristine ZnO photoanode, indicating the reduction of the resistance on the transport of charge. But the arc diameter was larger with the increasing thickness of the insulation overlayer (as shown in Figure S3 in the Supporting Information). In this work, the obtained results were fitted into the model of a RC circuit inserted in Fig. 5(a), in which R_ct_ is the interfacial charge-transfer resistance of the photoanode/electrolyte interface. Accordingly, R_ct_ was 39.78, 3.92 and 2.21 kΩ·cm^2^ for the pristine ZnO, ZnO/Au and ZAA-5 photoanodes. The smallest R_ct_ of ZAA-5 photoanode indicated the highest photocurrent density in line with the LSV curve shown in Fig. 3(a).

To further investigate the role of Au NPs and Al_2_O_3_ overlayer on the PEC performance, the photoluminescence (PL) experiments were carried out with 325 nm pulsed laser as excitation source. As shown in Fig. 5(b), the broad-band emission around 530 nm can be related to the recombination of photoexcited holes with electrons occupying the singly ionized oxygen vacancy (VO) and some structural defects at the surface of ZnO. And the relative intensity of the deep level (DL) emission can serve as an indicator for evaluating the electron-hole recombination. It’s obvious that the DL emission intensity decreased, demonstrating that the recombination rate of photogenerated electron-hole pairs decreased due to the surface passivation effect of Au NPs and Al_2_O_3_ overlayer.

**Figure 6:**
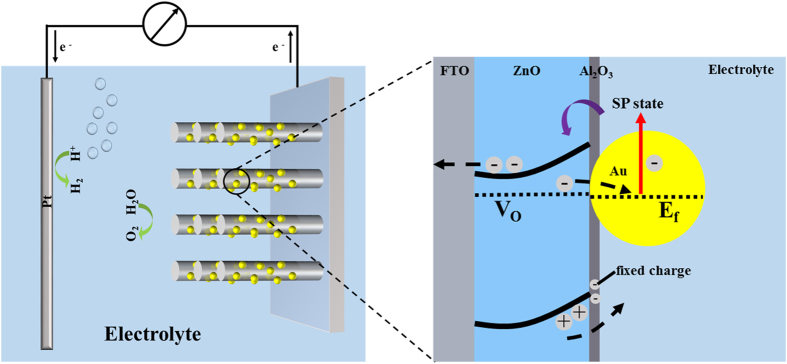
Scheme for the proposed mechanism of PEC water splitting and energy band diagram of ZnO/Au/Al_2_O_3_ photoanode.

Au atoms grown in oxygen vacancies on the surface of ZnO NRs could decrease the density of ionized oxygen vacancies, resulting in suppression of DL recombination^26^. Additionally, the energy level of defect states in ZnO (−4.99 eV) is close to the Au Fermi level (−5.3 eV), suggesting electrons in the defect states could flow into the Au Fermi level as shown in Fig. 6. And then under visible light illumination electrons by SPR excitation could transfer back to the conduction band of the ZnO NRs, resulting in an enhanced photocurrent.

As a dielectric medium, the ALD acquired Al_2_O_3_ overlayer could effectively decrease the surface defect states and thus increased the PEC performance of the photoanodes. Besides, the presence of negative fixed charge in Al_2_O_3,_ originated from the intrinsic (Al vacancies and O interstitial) and extrinsic (interstitial H) defects^27^,^28^, was demonstrated to reduce the recombination of Si^29^ and TiO_2_^30^. Such field-effect passivation is also suitable to explain the enhanced PEC performance in ZnO-based system. When UV illumination was applied, the photogenerated holes were trapped on the surface due to the presence of the negative charges, while the unpaired photogenerated electrons could transfer to Pt electrode via nanorods to participate in the water reduction, as shown in Fig. 6.

## Conclusions

In summary, Au NPs and Al_2_O_3_ overlayer were synergistically integrated into ZnO based photoanode for PEC water splitting. As a result, the increased absorption in visible region due to the SPR effect and the decreased photogenerated electron-hole recombination originated from the surface passivation, lead to a significantly enhanced PEC performance. The acquired PEC efficiency of ZnO/Au/Al_2_O_3_ (5 cycles) photoanodes under light illumination was 0.67%, 6.7 times that of pristine ZnO photoanode. The ZnO/Au/Al_2_O_3_ configuration showed great potential in hydrogen evolution, and further demonstrated the significance of synergetic effect of SPR and surface passivation in PEC system.

## Experimental Sections

### Fabrication of ZnO nanorods array

FTO were ultrasonically cleaned in acetone, ethanol and deionized (DI) water successively for 10 min, respectively. The colloidal seed solution of zinc acetate [Zn(CH_3_COO)_2_·2H_2_O] (0.5 M) was spin-coated onto the FTO substrate, and then annealed at 350 °C in air for 30 min. The ZnO nanorods array were prepared by the hydrothermal process in the aqueous solution containing 50 mM zinc nitrate hexahydrate [Zn(NO_3_)_2_·6H_2_O] and hexamethylenetetramine (HMTA) [(CH_2_)_6_N_4_] at 90 °C for 4 h. Finally, the substrate with products was repeatedly rinsed with deionized water for several times and annealed in air at 450 °C for 3 h to remove residual surface salts.

### Modification of Au nanoparticles

Under UV irradiation, the Au NPs were synthesized by photochemical deposition for 10 minutes in a 10 ml chloroauric acid (HAuCl_4_, 0.01%) solution with a pH = 7 adjusted by 0.1 M NaOH solution, 1 ml 1% polyvinyl alcohol (PVA, [C_2_H_4_O]n) and 0.5 ml methanol (CH_3_OH, 99.5%), followed by calcination at 350 °C in N_2_ for 30 minutes.

### Deposition of Al_2_O_3_ overlayer

Al_2_O_3_ coatings were performed with TALD-100A (kemicro) with 5, 10 and 20 cycles at a growth rate of ~1 Å/cycle. Argon (99.998%) was used as a carrier gas and employed at 25 sccm. A reactor pressure of 0.2 Torr was maintained at this argon gas flow. During the coating process, the system temperature was kept at 150 °C. Trimethylaluminum(Al(CH_3_)_3_) and DI water was pulsed into the reaction chamber separately with a pulsing time of 20 ms and 15 ms followed by 20 s N_2_ purging. Therefore, one deposition cycles involved 20 ms of trimethylaluminum pulse + 20 s of N_2_ purging + 15 ms of pulse H_2_O + 20 s of N_2_ purging. After deposition, the chamber was allowed to cool down naturally under N_2_ flow.

### Characterization of materials

The structures and morphologies of the electrode materials were characterized by X-ray diffraction (XRD) (Rigaku DMAX-RB, Cu Kα) and field emission scanning electron microscopy (FE-SEM) (FEI QUANTA 3D). The elements content of the samples was determined by Energy Dispersive X-Ray Spectroscopy (EDX). The formation of Au and Al_2_O_3_ on the ZnO nanorods and these species’ bonding characteristics were investigated by X-ray photoelectron spectroscopy (XPS, ESCALAB 250).

### Electrochemical characterization

The photoanode was fabricated by securing a copper wire on the exposed electric conductive parts of the FTO with a silver conducting paint. The substrate was subsequently sealed on all edge with epoxy resin except the active working area. All electrochemical measurements were performed in a three electrode mode with a photoanode as the working electrode, a coiled Pt wire as counter electrode and a Ag/AgCl reference electrode. 0.5 M Na_2_SO_4_ aqueous solution (with pH buffered to ~7.0) was adopted as the electrolyte. Electrochemical measurements were performed on an electrochemical workstation (Solartron SI 1287/SI 1260). All photoanodes were illuminated from the front side under AM 1.5 G illumination provided by a solar simulator (Oriel, 91159A). The white and visible light intensity was 50 and 40 mW/cm^2^ measured by a Si diode (Newport), respectively.

## Additional Information

**How to cite this article**: Liu, Y. *et al*. Synergistic Effect of Surface Plasmonic particles and Surface Passivation layer on ZnO Nanorods Array for Improved Photoelectrochemical Water Splitting. *Sci. Rep.* **6**, 29907; doi: 10.1038/srep29907 (2016).

## Supplementary Material

Supplementary Information

## References

[b1] Cho I. S. . Branched TiO_2_ nanorods for photoelectrochemical hydrogen production. Nano Lett. 11, 4978–4984 (2011). 21999403 10.1021/nl2029392

[b2] Wang G. . Significantly Enhanced Visible Light Photoelectrochemical Activity in TiO_2_ Nanowire Arrays by Nitrogen Implantation. Nano Lett. 15, 4692–4698 (2015). 26052643 10.1021/acs.nanolett.5b01547

[b3] Liu Y. . Design of sandwich-structured ZnO/ZnS/Au photoanode for enhanced efficiency of photoelectrochemical water splitting. Nano Res. 8, 2891–2900 (2015).

[b4] Bai Z. . 3D‐Branched ZnO/CdS Nanowire Arrays for Solar Water Splitting and the Service Safety Research. Adv. Energy Mat. , doi: aenm201501459 (2015).

[b5] Wang G. . Facile synthesis of highly photoactive alpha-Fe_2_O_3_-based films for water oxidation. Nano Lett. 11, 3503–3509 (2011). 21766825 10.1021/nl202316j

[b6] Wheeler D. . Nanostructured hematite: synthesis, characterization, charge carrier dynamics, and photoelectrochemical properties. Energy Environ. Sci. 5, 6682–6702 (2012).

[b7] Hou J. . High-performance p-Cu_2_O/n-TaON heterojunction nanorod photoanodes passivated with an ultrathin carbon sheath for photoelectrochemical water splitting. Energy Environ. Sci. 7, 3758–3768 (2014).

[b8] Wang M. . p–n Heterojunction photoelectrodes composed of Cu_2_O-loaded TiO_2_ nanotube arrays with enhanced photoelectrochemical and photoelectrocatalytic activities. Energy Environ. Sci. 6, 1211–1220 (2013).

[b9] Zhang Y. . Scanning probe study on the piezotronic effect in ZnO nanomaterials and nanodevices. Adv. Mat. 24, 4647–4655 (2012). 10.1002/adma.201104382 22488828

[b10] Shen Y. . Low-voltage blue light emission from n-ZnO/p-GaN heterojunction formed by RF magnetron sputtering method. Curr. Appl. Phys. 14, 345–348 (2014).

[b11] Kang Z. . Electronic structure engineering of Cu_2_O film/ZnO nanorods array all-oxide p-n heterostructure for enhanced photoelectrochemical property and self-powered biosensing application. Sci. Rep. 5, 7882 (2015). 25600940 10.1038/srep07882 PMC4298735

[b12] Sun Y. . High on-off ratio improvement of ZnO-based forming-free memristor by surface hydrogen annealing. ACS Appl. Mater. Inter. 7, 7382–7388 (2015). 10.1021/acsami.5b01080 25786156

[b13] Warren S. C. . Plasmonic solar water splitting. Energy Environ. Sci. 5, 5133–5146 (2012).

[b14] Zhang X. . 3D branched ZnO nanowire arrays decorated with plasmonic au nanoparticles for high-performance photoelectrochemical water splitting. ACS Appl. Mater. Inter. 6, 4480–4489 (2014). 10.1021/am500234v 24598779

[b15] Guo C. . Au@CdS Core–Shell Nanoparticles-Modified ZnO Nanowires Photoanode for Efficient Photoelectrochemical Water Splitting. Adv. Sci. 2, 1500135(2015). 10.1002/advs.201500135 PMC5115296 27980921

[b16] Wu M. . *In situ* growth of matchlike ZnO/Au plasmonic heterostructure for enhanced photoelectrochemical water splitting. ACS Appl. Mater. Inter. 6, 15052–15060 (2014). 10.1021/am503044f 25144940

[b17] Wang T. . Au nanoparticle sensitized ZnO nanopencil arrays for photoelectrochemical water splitting. Nanoscale 7, 77–81 (2015). 25113466 10.1039/c4nr03735a

[b18] Zhang Z. . Enhanced photoresponse of ZnO nanorods-based self-powered photodetector by piezotronic interface engineering. Nano Energy 9, 237–244 (2014).

[b19] Lu H. . Identifying the optimum thickness of electron transport layers for highly efficient perovskite planar solar cells. J. Mater. Chem. A 3, 16445–16452 (2015).

[b20] Lu H. . Interface Engineering through Atomic Layer Deposition towards Highly Improved Performance of Dye-Sensitized Solar Cells. Sci. Rep. 5, 12765 (2015). 26238737 10.1038/srep12765 PMC4523832

[b21] Hwang Y. . Photoelectrochemical properties of TiO_2_ nanowire arrays: a study of the dependence on length and atomic layer deposition coating. ACS Nano 6, 5060–5069 (2012). 22621345 10.1021/nn300679d

[b22] Le Formal F. . Passivating surface states on water splitting hematite photoanodes with alumina overlayers. Chem. Sci. 2, 737–743 (2011).

[b23] Lin C. . Enhanced performance of dye-sensitized solar cells by an Al_2_O_3_ charge-recombination barrier formed by low-temperature atomic layer deposition. J. Mater. Chem. 19, 2999–3003 (2009).

[b24] Kim W. . Promoting water photooxidation on transparent WO_3_ thin films using an alumina overlayer. Energy Environ. Sci. 6, 3732–3739 (2013).

[b25] Qiu Y. . Secondary branching and nitrogen doping of ZnO nanotetrapods: building a highly active network for photoelectrochemical water splitting. Nano Lett. 12, 407–413 (2012). 22149105 10.1021/nl2037326

[b26] Park S. . Enhanced photoluminescence of Au-functionalized ZnO nanorods annealed in a hydrogen atmosphere. Luminescence 147, 5–8 (2014).

[b27] Hoex B. . On the c-Si surface passivation mechanism by the negative-charge-dielectric Al_2_O_3_. J. Appl. Phys. 104, 113703 (2008).

[b28] Matsunaga K. . First-principles calculations of intrinsic defects in Al_2_O_3_. Phys. Rev. B 68, 085110 (2003).

[b29] Terlinden N. . Role of field-effect on c-Si surface passivation by ultrathin (2–20 nm) atomic layer deposited Al_2_O_3_. Appl. Phys. Lett. 96, 112101 (2010).

[b30] Gui Q. . Enhanced photoelectrochemical water splitting performance of anodic TiO_2_ nanotube arrays by surface passivation. ACS Appl. Mater. Inter. 6, 17053–17058 (2014). 10.1021/am504662w 25198058

