# Supplementary material for: Synergistic Effect of Surface Plasmonic particles and Surface Passivation layer on ZnO Nanorods Array for Improved Photoelectrochemical Water Splitting
Source: Sci Rep. 2016 Jul 21;6:29907. doi: 10.1038/srep29907 (PMC4956743; doi:10.1038/srep29907)
Supplement: Supplementary Information [file srep29907-s1.doc]

**Supplementary Information**

**Synergistic Effect of Surface Plasmonic particles and Surface Passivation layer on ZnO Nanorods Array for Improved Photoelectrochemical Water Splitting**

Yichong Liu,^1^ Xiaoqin Yan,^1^ Zhuo Kang,^1^ Yong Li,^1^ Yanwei Shen,^1^ Yihui Sun^1^, Li Wang^2^ and Yu Zhang^1,3^

1. State Key Laboratory for Advanced Metals and Materials, School of Materials Science and Engineering, University of Science and Technology Beijing, Beijing 100083, China.

2. Civil and Environment Engineering school, University of Science and Technology Beijing, Beijing 100083, China.

3. The Beijing Municipal Key Laboratory of New Energy Materials and Technologies, University of Science and Technology Beijing, Beijing 100083, China.


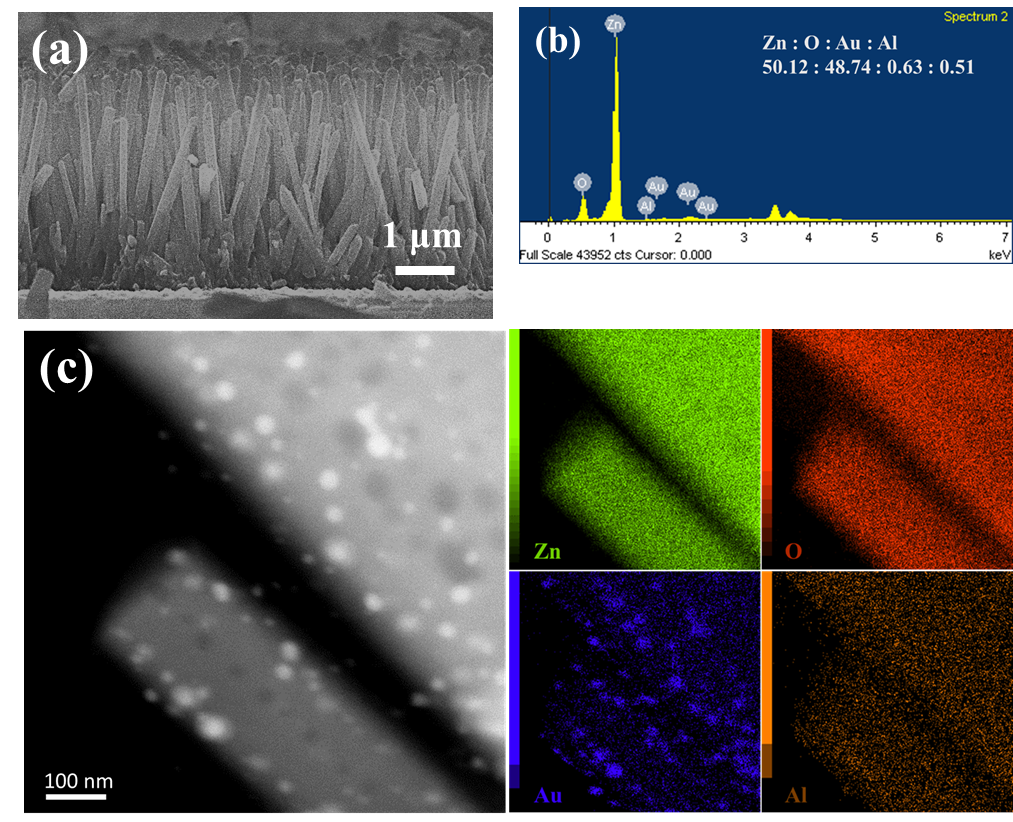


Figure S1 (a) cross-sectional-view SEM image of ZnO nanorods array. (b) and (c) EDS spectra of ZnO/Au/Al_2_O_3_ (5 cycles).


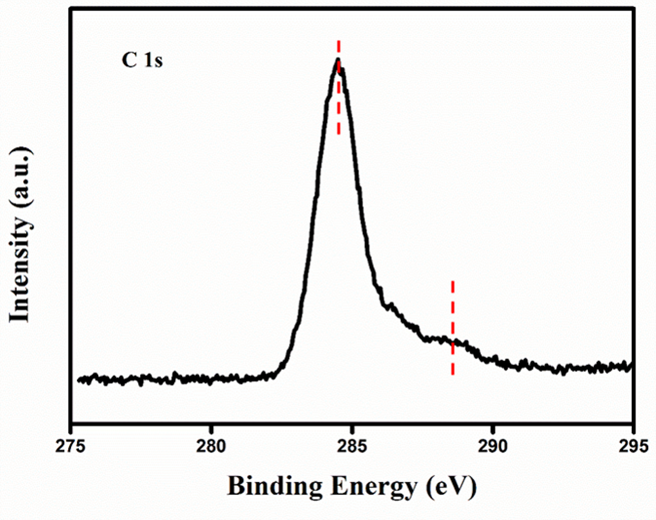


Figure S2 XPS spectra of C 1s core level of the ZnO/Au/Al_2_O_3_. The strong sharp peak located at 284.5 eV can be attributed to free carbon from contamination of the sample. The higher binding-energy peak located at 288.6 eV can be ascribed to be the bsorbed C=O or C-O from the carbon-contained purities.


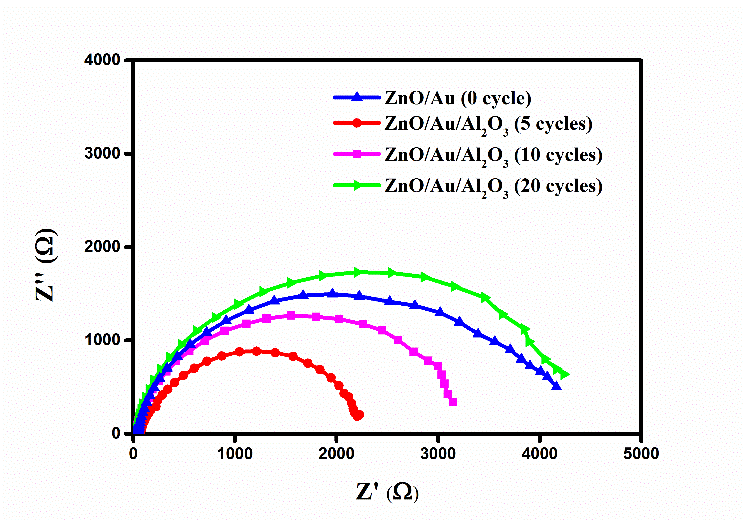


Figure S3 (a) LSV curves and (b) nyquist plots of electrochemical impedance spectra recorded for the ZnO/Au/Al_2_O_3_ photoanodes with various cycles.
